# Predicting motor learning performance from Electroencephalographic data

**DOI:** 10.1186/1743-0003-11-24

**Published:** 2014-03-04

**Authors:** Timm Meyer, Jan Peters, Thorsten O Zander, Bernhard Schölkopf, Moritz Grosse-Wentrup

**Affiliations:** 1 Department Empirical Inference, Max Planck Institute for Intelligent Systems, Tübingen, Germany; 2 Technische Universität Darmstadt, Intelligent Autonomous Systems Group, Darmstadt, Germany; 3 Team PhyPA, Department for Biopsychology and Neuroergonomics, Berlin Institute of Technology, Berlin, Germany

**Keywords:** Visuomotor integration and learning, Motor learning, Brain-computer interface, BCI, EEG, Performance prediction

## Abstract

**Background:**

Research on the neurophysiological correlates of visuomotor integration and learning (VMIL) has largely focused on identifying learning-induced activity changes in cortical areas during motor execution. While such studies have generated valuable insights into the neural basis of VMIL, little is known about the processes that represent the current state of VMIL independently of motor execution. Here, we present empirical evidence that a subject’s performance in a 3D reaching task can be predicted on a trial-to-trial basis from pre-trial electroencephalographic (EEG) data. This evidence provides novel insights into the brain states that support successful VMIL.

**Methods:**

Six healthy subjects, attached to a seven degrees-of-freedom (DoF) robot with their right arm, practiced 3D reaching movements in a virtual space, while an EEG recorded their brain’s electromagnetic field. A random forest ensemble classifier was used to predict the next trial’s performance, as measured by the time needed to reach the goal, from pre-trial data using a leave-one-subject-out cross-validation procedure.

**Results:**

The learned models successfully generalized to novel subjects. An analysis of the brain regions, on which the models based their predictions, revealed areas matching prevalent motor learning models. In these brain areas, the *α*/*μ* frequency band (8–14 Hz) was found to be most relevant for performance prediction.

**Conclusions:**

VMIL induces changes in cortical processes that extend beyond motor execution, indicating a more complex role of these processes than previously assumed. Our results further suggest that the capability of subjects to modulate their *α*/*μ* bandpower in brain regions associated with motor learning may be related to performance in VMIL. Accordingly, training subjects in *α*/*μ*-modulation, e.g., by means of a brain-computer interface (BCI), may have a beneficial impact on VMIL.

## Background

Motor learning of novel kinematic and/or dynamic environments can be categorized by learning phase and learning form [1]. The temporal course of motor learning is oftentimes divided into three phases, an early- (slow performance, close sensory guidance), an intermediate- (gradual learning, increase in speed) and an advanced phase (skillful and automatized movements) [2]. In the early stage of motor learning prefrontal areas play a key role, especially the dorsolateral frontal cortex and the right prefrontal cortex [3-6]. This key role might be due to the fact that early learning is closely related to attention and relies on explicit working memory and forming new associations between visual cues and motor commands [7]. Other areas involved in early stages of motor learning include (pre-)motor areas [5,7] and superior-posterior parietal cortex [8]. In later stages, prefrontal activation shifts more to the left hemisphere [6,7]. This left-hemispheric dominance appears to be independent of the side used for training [9]. Furthermore, the learning process can be categorized into two forms: explicit learning, in which subjects consciously try to learn a task relying on previous experiences, and implicit learning, which takes place unintentionally and unconsciously.

To date, one widely accepted model of motor learning is the one proposed by Hikosaka and colleagues [10]. This model comprises two parallel loop circuits, one responsible for learning spatial features (frontoparietal–associative striatum–cerebellar circuit), and the other one responsible for learning motor features (motor cortex–sensorimotor striatum–cerebellar circuit). Transformations between the two loops take place in the supplementary motor area (SMA), the pre-SMA and the premotor cortices.

In this article, we investigate whether the involvement of these areas in motor learning is restricted to periods of actual motor execution, or if they also represent the current state of motor learning when subjects are either at rest or are preparing for an upcoming movement. We present results of an EEG study on explicit learning of a sensorimotor task, and provide empirical evidence that cortical structures known to be involved in motor learning do indeed provide information about the actual progress of motor learning, i.e. they predict the precision of an upcoming movement. We discuss the implications of these findings for motor learning in general and the use of brain-computer interfaces (BCIs) for motor rehabilitation in particular.

## Methods

### Subjects

Six healthy subjects (3 male, 3 female; mean age 29.5±4.5), recruited from the local student body, participated in the present study. All subjects were right-handed and thus conducted the study with their right arm. All subjects except subject three were naive to the task. Subject three had participated in a similar experiment with two dimensional reaching movements. All subjects gave informed consent in accordance with guidelines set by the Max Planck Society. The paradigm and experimental setup of this study have been approved by an ethics committee of the Max Planck Society.

### Study design

**Figure 1:**
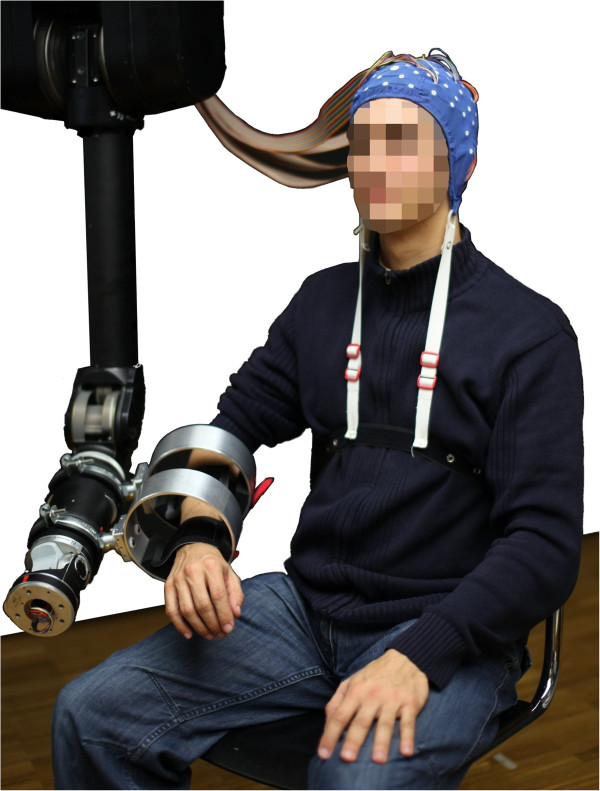
Subject wearing an EEG-cap while being attached to the seven DoF Barrett WAM arm (subject consented to this image being used in this publication).

The subject’s right arm was attached to a seven degrees of freedom (DoF) robotic arm (Figure 1) facing a feedback screen at a distance of approximately 1.5 meters. Due to the robotic arm’s DoF, the subjects were able to perform a large variety of natural movements. The robot compensated gravity for its own weight, therefore the subject required only negligible forces to move his and the robot’s arm. In this study the robotic arm was used in a purely passive way, whereas the system is designed in a way that the robot can disturb, influence or support movements (see [11] for a more detailed description of the robotic setup).

The goal of each trial was to move the robot arm to reach a target visualized in 3D on a computer screen and thereafter back to the initial starting position. For each trial, the target was chosen from a set of pre-defined targets. The screen continuously displayed the current position of the robotic arm’s end-effector and the target position. Each subject performed 200 trials divided into four blocks of 50 trials, interleaved with a brief one minute intermission. EEG data was continuously recorded during the experiment.

### Trial design

**Figure 2:**
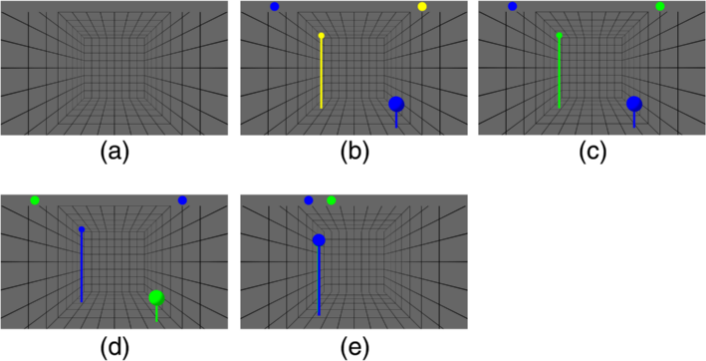
**Visual feedback.(a)** Feedback shown in the baseline phase. **(b)** Feedback shown in the planning phase. **(c)** Feedback shown in the go phase. **(d)** Feedback shown in the return to start phase. **(e)** The figure shows a state in which the depth bar provides information which is otherwise not easy to see.

Table 1 provides an overview over the four phases that each trial consisted of. An overview over the visualization is provided in Figure 2. Each trial started with the *baseline* phase, for which the subject was instructed to do nothing and no feedback was shown (c.f. Figure 2(a)). After 5 seconds, the phase switched to the *planning* phase for which the subject was instructed to plan the movement, but not yet move. This phase change was indicated by displaying the current end-effector’s position as blue ball and the target as yellow ball (c.f. Figure 2(b)). The phase lasted 2.5–4 seconds, with the duration chosen randomly from an uniform distribution. The experiment then continued with the *go* phase, which was indicated by switching the target’s color from yellow to green (c.f. Figure 2(c)). The subject was instructed to bring the current hand position in congruence with the target position, i.e. reach for the target. A reaching movement was considered complete when the subject moved the end-effector within 1.5 cm of the target location, or if the subject exceeded a ten seconds time limit. In either case, the green ball at the target position disappeared and was replaced by a green ball at the initial starting position of the end-effector (c.f. Figure 2(d)). This event started the last phase - *return to start* - for which the subject was instructed to return to the starting position. When the subject moved the end-effector to within 4 cm of the initial position or a time limit of ten seconds ran out, the robot arm gently pulled the end-effector to its precise starting position for the next trial.

**Table 1:** Experiment phases

**Phase**	**Duration**	**Instruction**	**Visualization**
Baseline	5 secs	Do nothing	Neither target nor current position was shown
Planning	2.5–4 secs	Plan path	Current position was shown as blue ball, target position as yellow
Go	Max 10 secs	Reach target	Current position was shown as blue ball, target position as green
Return to start	Max 10 secs	Return to start	current position was shown as blue ball, initial position as green

This table describes the phases of the experiment regarding their name, duration, the instruction provided to the subject and the visual feedback provided.

In each trial, a different target location was chosen from a sphere located in front of the subject. In order to determine a range of reachable targets, while considering the subject’s individual physical differences, each subject determined the center and radius of the sphere prior to initiation of the first trial by moving their arm to multiple comfortable positions in front of their body. The sphere was defined as the minimum sphere that enclosed 90% of all the positions visited while the subject moved between these comfortable positions. In this study, the radii varied from 5–9 cm.

### Visualization

The visual feedback consisted of a virtual box in which the current end-effector’s position and the target position were displayed as colored balls (c.f. Figure 2). The bar at the top of the screen provided information about the depth of the balls. Both balls were plotted on this bar according to their z coordinate. A position farther on the left on the bar indicated a position closer to the subject. This bar was added to enhance depth-perception when the target and current position overlapped on the screen (c.f. Figure 2(e)). This problem originates in projecting a three dimensional task onto a two dimensional fixed plane. For the same reason poles were added to the balls. These poles provided information about the projected location of the balls on the ground plane, thus making it easier to estimate depth and distances.

### Data acquisition

Throughout the study, a 120-channel EEG was recorded at 1 kHz sampling rate, using active EEG electrodes and a QuickAmp amplifier (BrainProducts, Gilching, Germany). Electrodes were placed according to the extended 10-20 system, with Cz as the initial reference electrode. All data were re-referenced to common average reference offline.

To track each subject’s learning process over the course of the experiment, the normalized time-to-target (TTT) for each trial was computed, i.e. the time required from the instruction to initiate the movement to reaching the target, divided by the distance from starting position to target position.

### Data analysis

In this section, we describe our data analysis, which was conducted to examine whether TTT can be predicted from EEG signals originating in the baseline or planning phase. We employed a random forest model and a leave-one-subject-out cross-validation for this purpose.

#### Time to target prediction

**Figure 3:**
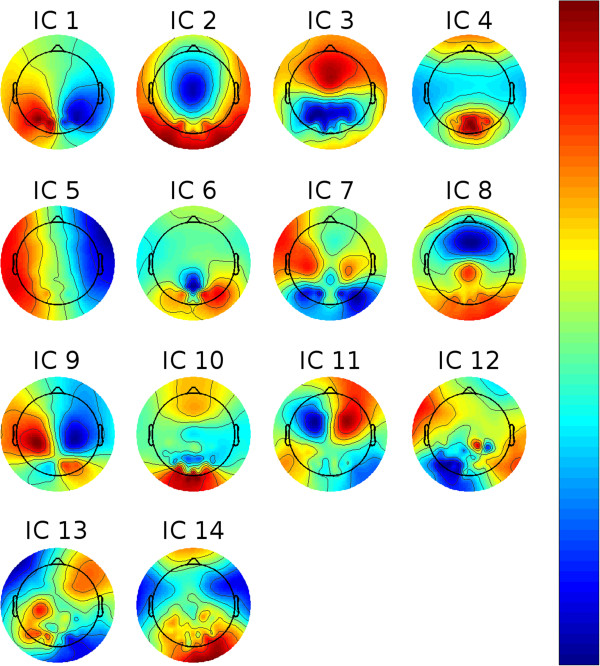
**All non-rejected ICs.** The figure shows all ICs that were kept after rejecting non cortical ICs.

In the following, we investigate whether TTT can be predicted on a trial-to-trial basis from EEG recorded in the upcoming target’s planning or baseline phase. To do so, we separated the data into group-wise (ideally) statistically independent components (ICs). This was done by first high-pass filtering each subject’s raw data at 3 Hz using a third-order Butterworth filter. The data of all subjects were pooled and reduced to 64 principal components before applying a second-order blind identification algorithm (SOBI) [12]. We inspected each IC manually and rejected those which were not of cortical origin or did not contain EEG-like spectral densities. The topographies of the remaining ICs are shown in Figure 3. We computed log-bandpower of each non-artifactual IC in each trial in five frequency bands (based on the raw data using a FFT in conjunction with a Hann window): *δ* (0.1–4 Hz), *θ* (4–7 Hz), *α*/*μ* (8–14 Hz), *β* (20–30 Hz), and *γ* (55–85 Hz). We low-pass filtered these bandpowers in the trial domain with.1 radians since we were mostly interested in slow changes in contrast to fast variations (see *Results and discussion* section).

These bandpowers served as input variables to a random forest trained on five subjects, to predict the normalized TTT on the remaining subject. With 14 ICs and five different frequency bands, this random forest model operates on a 70 dimensional feature space. Cross-validation within a subject requires data to be independent and identically distributed (IID) [13], which is not the case for movement performance data. We therefore employed a leave-one-subject-out cross-validation instead. This was done in turn for all subjects.

Since the input variables are low-pass filtered, our model cannot capture high-frequency changes. Thus, quality estimation of the prediction using standard *R*^2^, the coefficient of determintation, would be underrated. To provide a better quality estimate, the following modification of *R*^2^ was used. Let *R*^2^(*x*,*y*) denote the usual definition of *R*^2^ with the observed values *x* and the modeled values *y*, and let *LP*(*x*,*c*) denote variable *x* low-pass filtered with a cut-off frequency of *c* radians. *R*^2^(*x*,*LP*(*x*,*c*)) reflects the amount of variance that can be explained by using only the low-frequency components of *x* and thus represents the maximum achievable value for a random forest model based on frequencies less or equal than *c* radians. We then define

(1) $$R_{\text{mod}}^{2}(x,y)=\frac{R^{2}(x,y)}{R^{2}(x,\text{LP}(x,c))}.$$

Thus $R_{\text{mod}}^{2}$ ranges from −*∞* to 1, where a value less than 0 means that additional variance was introduced, e.g., by using a completely random prediction. All values over 0 are desirable, with 1 being the best achievable value, i.e. explaining all variance that can be explained by low frequencies. The specific value of *c* used is explained in chapter *Results and discussion*. We calculated $R_{\text{mod}}^{2}$ between predicted and actual TTT, and tested group level significance with a permutation test. For this test, the trial order was permuted independently for each subject, and the average of $R_{\text{mod}}^{2}$ over all subjects was calculated. This was repeated 10,000 times and significance was estimated by using the relative position of the real average $R_{\text{mod}}^{2}$ in comparison to the permutation based $R_{\text{mod}}^{2}$ values.

### Model interpretability

Although random forests are based upon decision trees, random forests lack the interpretability of these. As a consequence, Breiman devised a measure that reflects the importance of a variable for an accurate prediction [14]. Computing variable importance is based on measuring the accuracy drop in case the values of one variable are permuted.

After using variable importance to analyze which input variables have a large influence on the prediction, one can use the learned model to see how these variables affect the predictions, as described in the following approach. In the context of this study, a variable *v* refers to the bandpower of one IC in one specific frequency band. A reasonable range of values to analyze is defined by the minimum and maximum value that was encountered in the training data for the analyzed variable *v*. Any value outside this range has the same effect on the prediction as the minimum or maximum value. To determine the average effect of a variable *v* on the prediction the following steps are performed: (1) in all training data, replace the measured value of variable *v* by $\hat{v}$, (2) use the model to predict the output for the modified data, (3) the average output provides an estimate of the effect of setting *v* to $\hat{v}$ on the prediction. Once a sufficiently sized subset of the range is analyzed, this provides an assessment of the variable’s influence on the prediction.

In the context of this study, this approach was used to estimate how frequency-specific bandpower changes in cortical areas influence the prediction of TTT.

## Results and discussion

**Figure 4:**
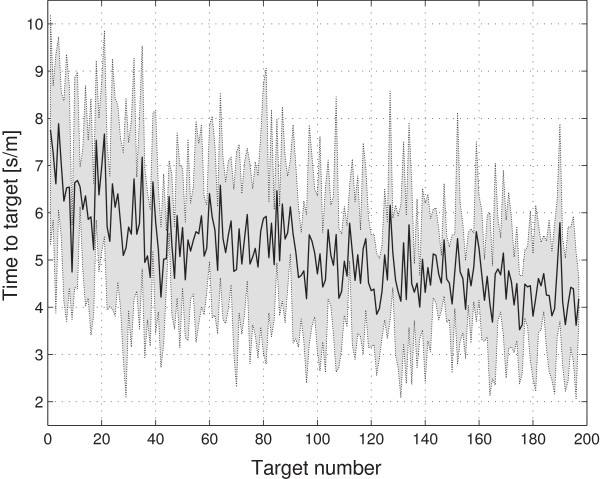
**Mean and Standard Deviation of TTT.** Mean and standard deviation of the changes in time-to-target across the experimental session for the six subjects.

**Figure 5:**
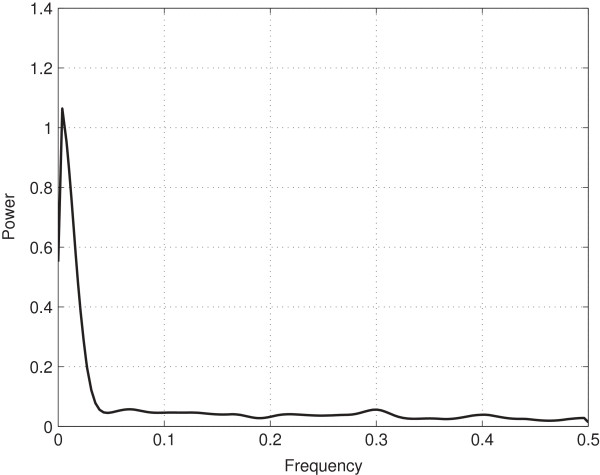
**PSD of Mean TTT.** This figure shows the PSD of the mean TTT.

**Figure 6:**
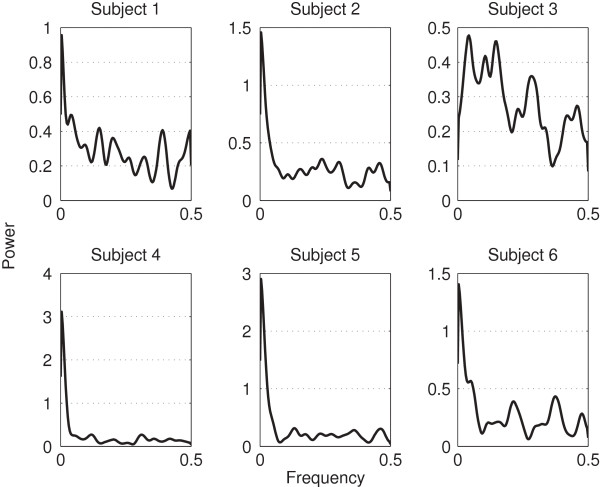
**PSD of each subject.** This figure shows the PSD of each subjects’ TTT.

**Figure 7:**
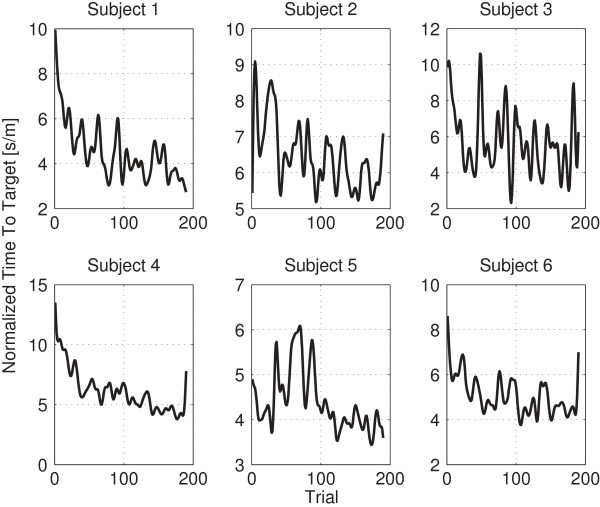
**Normalized TTT of each subject.** This figure shows each subjects’ TTT lowpass filtered at.1 radians and normalized.

The average time a subject needed to complete the experiment was 60.17 minutes with a standard deviation of 1.18 minutes. We observed a continuous decline in TTT over the course of the experiment, reflecting successful VMIL processes (Figure 4). This trend is captured by the low frequencies of the TTTs’ power spectral density (PSD) (Figure 5). After analyzing the power spectral densities of each subject’s TTT (Figure 6), we concluded that the most important frequency components exist below.1 radians. This value was then used as value *c* for low-pass filtering the TTT, as described in the data analysis section. Figure 7 shows the TTT of each subject low-pass filtered at.1 radians. We want to point out that due to low-pass filtering the term *predicting* could be misleading. For the purpose of text flow and as this term is oftentimes just referring to a model’s output, we will stick to the term prediction.

### Time to target prediction

Table 2 shows $R_{\text{mod}}^{2}$ for all subjects with a model trained on data from the baseline or the planning phase, as previously described. Significance values on a group level are provided, which were computed as described in the previous section. Subjects three and five show low $R_{\text{mod}}^{2}$ values for both phases. Subject five told us after the experiment that he was tired during the experiment and showed signs of fatigue. This information is in congruence with his TTT (c.f. Figure 7). Since his prediction model was based on the other subject’s TTT and brain signals - and they did not show signs of fatigue - this might be the reason for his low $R_{\text{mod}}^{2}$ value. Subject three had previous experience with this kind of experiment and showed only little signs of improvement after a phase of familiarization. This existing experience might explain his low $R_{\text{mod}}^{2}$ value. The group level permutation test rejected the null-hypothesis that the temporal structure of the features does not provide any information on the current state of VMIL for data from the baseline phase (*p*<0.001) as well as from the planning phase (*p*<0.001).

**Table 2:** Prediction quality estimation

**Subject**	**Baseline**	**Planning**
1	0.03	0.19
2	0.23	0.45
3	-0.13	-0.53
4	0.10	0.16
5	-0.10	-0.09
6	0.04	0.18
Group median	0.03 (*p*-value <0.001)	0.17 (*p*-value <0.001)

This table provides the $R_{\text{mod}}^{2}$ value for models trained with leave-one-subject-out cross-validation on data from the respective phase.

**Figure 8:**
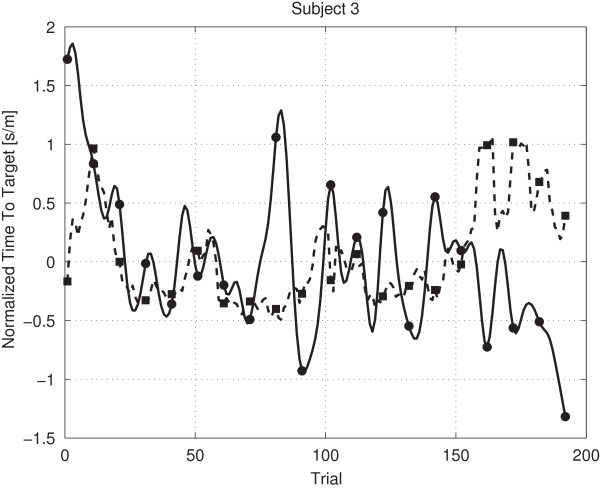
**Prediction subject 3 planning.** The figure shows the predicted values (dashed line) and the lowpass filtered real values (solid line) for subject 3 (planning phase, $R_{\text{mod}}^{2}$ is -0.53).

**Figure 9:**
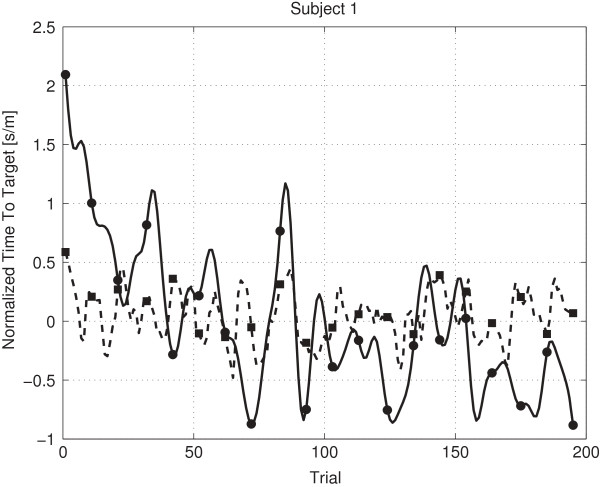
**Prediction subject 1 baseline.** The figure shows the predicted values (dashed line) and the lowpass filtered real values (solid line) for subject 1 (baseline phase, $R_{\text{mod}}^{2}$ is 0.03).

**Figure 10:**
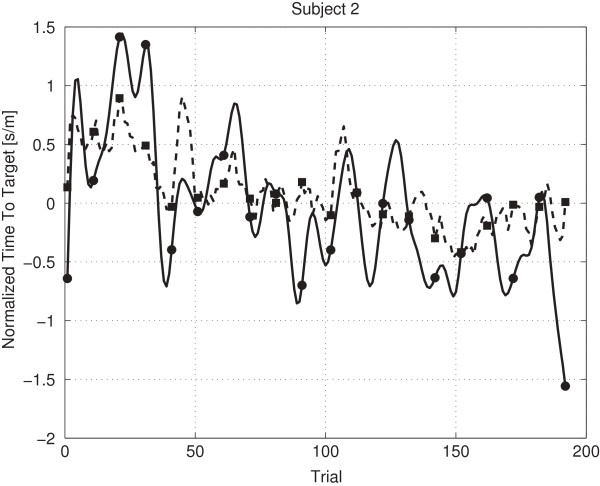
**Prediction Subject 2 Planning.** The figure shows the predicted values (dashed line) and the lowpass filtered real values (solid line) for subject 2 (planning phase, $R_{\text{mod}}^{2}$ is 0.45).

Figures 8, 9 and 10 provide examples of a low (-0.53), near zero (0.03) and high (0.45) $R_{\text{mod}}^{2}$ value. Figure 8 shows that already a small amount of values contradicting the general trend leads to a low $R_{\text{mod}}^{2}$ value. Figure 9 shows a prediction oscillating around the mean value, corresponding to a $R_{\text{mod}}^{2}$ value near zero. Figure 10 shows an example for a moderately high, positive $R_{\text{mod}}^{2}$ value.

### Source localization and relation to existing motor learning models

**Figure 11:**
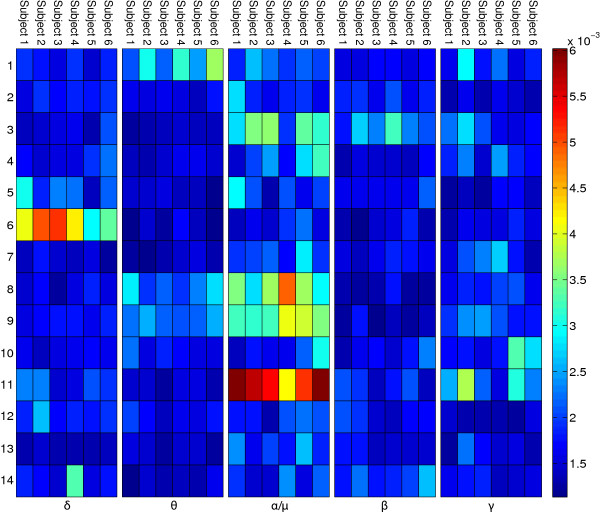
**Importance baseline.** The figure shows the variable importance values for all ICs in the baseline phase per subject.

**Figure 12:**
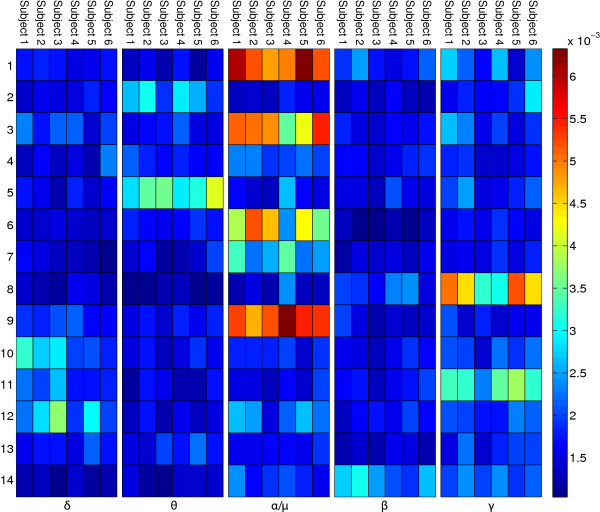
**Importance planning.** The figure shows the variable importance values for all ICs in the planning phase per subject.

In order to identify cortical areas relevant for TTT-prediction, the variable importance values of the random forests were investigated for each subject’s model (Figures 11 and 12).

**Figure 13:**
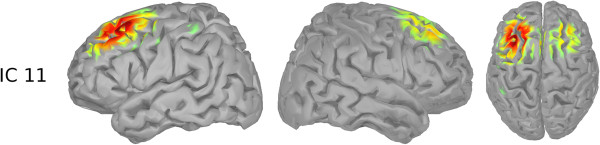
**Source localization IC 11.** The figure shows the source localization of IC 11 (left view, right view, top view).

**Figure 14:**
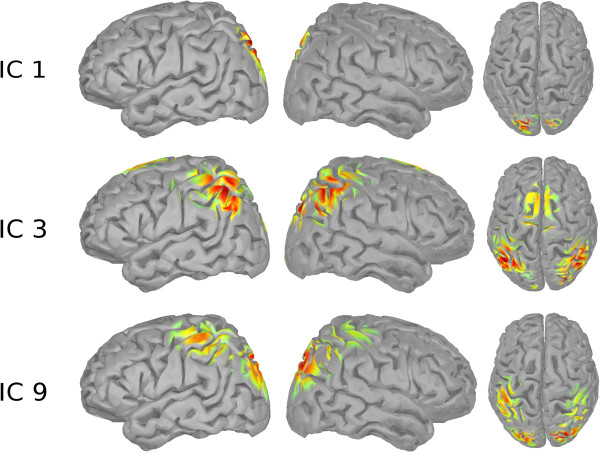
**Source localization ICs 1, 3, 9.** The figure shows the source localizations of ICs 1, 3, 9 (left view, right view, top view).

For the baseline phase, the *μ* band of IC 11 shows the highest importance values, consistent across subjects. The corresponding ICs’ topographies (cf. Figure 4) were projected back to the cortical level using the BrainStorm toolbox [15]. For this purpose a distributed source model with minimum-norm estimation was selected, based on standard electrode locations and a standard head model. Figure 13 shows the source localization result for IC 11. This IC contains strong weights in prefrontal areas, possibly focused in the dorsolateral prefrontal cortex. It is commonly reported that the dorsolateral prefrontal cortex is involved in the initial stages of explicit motor learning, due to its role in sensorimotor association and working memory [4,6]. Figure 14 shows the source localization results for ICs 1, 3 and 9, which are the most relevant ICs in the planning phase. These ICs are primarily localized to parietal cortex, but also exibit activity in preSMA, SMA, primary somatosensory cortex, associative visual cortex (V3, V4, V5), prestriate cortex (V2) and somatosensory association cortex. Since the planning phase provided information about the next target, the contribution of these regions to the prediction is in congruence with Hikosaka’s model, stating that preSMA and SMA are responsible for spatiomotor conversion processes. The activity in the posterior parietal cortex confirms the finding that this region is involved in motor planning [8].

Due to pooling the EEG data before separating ICs one could argue that a small subset of subjects could dominate certain ICs and therefore distort group effects. This argument is weakened by the fact that the most important ICs are consistent across subjects, as seen in Figures 11 and 12.

### Feature influence on prediction

**Figure 15:**
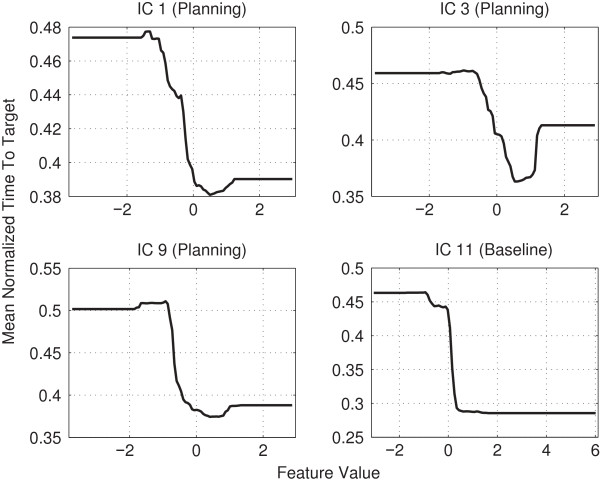
**Change of TTT when altering*α*/*μ* band for ICs 1, 3, 9, 11.** This figure shows the effect a change of the bandpower in the *α*/*μ* band has on the TTT prediction. ICs 1, 3 and 9 are in regard to the planning phase, IC 11 is in regard to the baseline phase.

For both the baseline- and the planning phase, the *α*/*μ* frequency band was found to be most relevant. To analyze the relation between this band and TTT prediction, we examined the effect of changing the bandpower in the aforementioned ICs as described in section *Model interpretability*. The results are shown in Figure 15. They indicate that enhanced bandpower in this frequency band in the previously mentioned regions is related to better movement performance.

## Conclusions

Previous research on VMIL concentrated on investigating learning-induced changes in brain activity during motor execution. In our study we presented empirical results indicating that motor performance can be predicted from pre-trial EEG signals, thus identifying brain regions not only actively involved in motor learning, but furthermore providing information about the current learning progress. Areas found to be involved include the dorsolateral prefrontal cortex, preSMA, SMA, primary somatosensory cortex, V2, V3, V4, V5 and somatosensory association cortex. These results might provide starting points for enhancing motor learning and increasing motor rehabilitation performance, e.g., by neurofeedback [16] or with a direct stimulation as is the case in transcranial direct current stimulation [17].

### Implications for motor imagery based BCI stroke rehabilitation

While initially conceived as communication devices, brain-computer interfaces (BCIs) have recently attracted attention as potential tools for stroke rehabilitation [16,18-20]. Here, the central idea is to train patients in modulating sensorimotor-rhythms (SMRs) by real-time neurofeedback, as the extent of SMR-modulation has been found to correlate with stroke severity [21]. Our results provide further support for this novel form of therapy, as they indicate that training subjects in SMR-modulation may have a beneficial impact on their VMIL skills (cf. Figure 14 (IC 9) and Figure 15). We conjecture that subjects with better VMIL skills require less training to relearn a disturbed mapping between movement goals and motor commands, potentially resulting in enhanced post-stroke motor learning. Our results further suggest that BCI studies on stroke rehabilitation should extend their focus beyond SMR-training. As we found parietal areas to be most useful for VMIL prediction, stroke subjects may also benefit from neurofeedback training that aims to enhance *α*/*μ*-rhythms originating in parietal cortex.

## Abbreviations

BCI: Brain-computer interface; DoF: Degrees of freedom; EEG: Electroencephalography; FFT: Fast fourier transform; IC: Independent component; ICA: Independent component analysis; IID: Independent and identically distributed; PSD: Power spectral density; SMA: Supplementary motor area; SMR: Sensori-motor rhythm; TTT: Normalized time to target; VMIL: Visuomotor integration and learning.

## Competing interests

The authors declare that they have no competing interests.

## Authors’ contributions

TM participated in the design of the study, conducted the study, performed the data analysis and drafted the manuscript. JP contributed to the experimental design as well as to the setup of the robotic system and participated in writing the manuscript. TOZ contributed to the experimental design. BS was involved in planning and conception of experimental paradigm and evaluation. MGW initiated the study, contributed to the experimental design and the data analysis, and participated in writing the manuscript. All authors read and approved the final manuscript.
